# Blockade of Transient Receptor Potential Vanilloid 4 Enhances Antioxidation after Myocardial Ischemia/Reperfusion

**DOI:** 10.1155/2019/7283683

**Published:** 2019-06-16

**Authors:** Qiongfeng Wu, Kai Lu, Zhaoyang Zhao, Binbin Wang, Huixia Liu, Shaoshao Zhang, Jie Liao, Yu Zeng, Qian Dong, Ning Zhao, Bing Han, Yimei Du

**Affiliations:** ^1^Department of Cardiology, Union Hospital, Tongji Medical College, Huazhong University of Science and Technology, Wuhan 430022, China; ^2^Research Center of Ion Channelopathy, Union Hospital, Tongji Medical College, Huazhong University of Science and Technology, Wuhan 430022, China; ^3^Institute of Cardiology, Union Hospital, Tongji Medical College, Huazhong University of Science and Technology, Wuhan 430022, China; ^4^Key Lab for Biological Targeted Therapy of Education Ministry and Hubei Province, Union Hospital, Tongji Medical College, Huazhong University of Science and Technology, Wuhan 430022, China; ^5^Grade 2017, School of pharmacy, South-Central University for Nationalities, 182 Minyuan Road, Wuhan 430074, China; ^6^Department of Cardiology, Xuzhou Central Hospital, Jiefang Nan Lu 199, Xuzhou, Jiangsu 221009, China

## Abstract

Antioxidative stress provides a cardioprotective effect during myocardial ischemia/reperfusion (I/R). Previous research has demonstrated that the blockade of transient receptor potential vanilloid 4 (TRPV4) attenuates myocardial I/R injury. However, the underlying mechanism remains unclear. The current study is aimed at investigating the antioxidative activity of TRPV4 inhibition and elucidating the underlying mechanisms *in vitro* and *ex vivo*. We found that the inhibiting TRPV4 by the selective TRPV4 blocker HC-067047 or specific TRPV4-siRNA significantly reduces reactive oxygen species (ROS) and methane dicarboxylic aldehyde (MDA) levels in H9C2 cells exposed to hypoxia/reoxygenation (H/R). Meanwhile, the activity of antioxidative enzymes, particularly superoxide dismutase (SOD) and glutathione peroxidase (GSH-Px), is enhanced. Furthermore, after H/R, HC-067047 treatment increases the expression of P-Akt and the translocation of nuclear factor E2-related factor 2 (Nrf2) and related antioxidant response element (ARE) mainly including SOD, GSH-Px, and catalase (CAT). LY294002, an Akt inhibitor, suppresses HC-067047 and specific TRPV4-siRNA-induced Nrf2 expression and its nuclear accumulation. Nrf2 siRNA attenuates HC-067047 and specific TRPV4-siRNA-induced ARE expression. In addition, treatment with LY294002 or Nrf2 siRNA significantly attenuates the antioxidant and anti-injury effects of HC-067047 *in vitro*. Finally, in experiments on isolated rat hearts, we confirmed the antioxidative stress roles of TRPV4 inhibition during myocardial I/R and the application of exogenous H_2_O_2_. In conclusion, the inhibition of TRPV4 exerts cardioprotective effects through enhancing antioxidative enzyme activity and expressions via the Akt/Nrf2/ARE pathway.

## 1. Introduction

Ischemia/reperfusion (I/R) injury is the leading factor that aggravates myocardial dysfunction and cardiomyocyte death after cardiac surgery and myocardial infarction [1]. The mechanisms of I/R injury are complex, mainly including intracellular Ca^2+^ overload and excessive reactive oxygen species (ROS) production [2, 3]. It is noteworthy that the accumulation of intracellular ROS can lead to apoptotic cell death [4] and has a direct effect on myocardial structure and function [5]. Therefore, the activation of endogenous antioxidant defense likely ameliorates myocardial I/R injury.

Nuclear factor-erythroid 2-related factor 2 (Nrf2), as a redox-sensitive transcription factor, regulates the cellular antioxidant defense system by binding to antioxidant response elements (AREs) in their promoter regions [6]. After bonding with AREs in the nucleus, Nrf2 can trigger the expression of antioxidative genes, such as superoxide dismutase (SOD), catalase (CAT), glutathione S-transferase (GST), glutathione peroxidase (GPx), NAD (P) H:quinone oxidoreductase 1 (NQO1), and heme oxygenase 1 (HO-1) [7, 8]. Notably, the phosphorylation of Akt has been shown to alleviate oxidative stress injury through promoting the activation of Nrf2/ARE signaling [9, 10]. Therefore, Akt/Nrf2/ARE might be a valuable therapeutic target for the prevention of myocardial I/R-induced oxidative stress and damage.

Ca^2+^ permeable transient receptor potential vanilloid 4 (TRPV4) acts as a polymodal detector to sense microenvironmental changes in tissues, such as hypotonic stimulation, cell swelling, warm temperature (>24-37°C), and the presence of endogenous arachidonic acid metabolites [11, 12]. These changes commonly occur in ischemic tissues or during tissue recovery after the restoration of blood perfusion. The upregulation of TRPV4 expression has been observed in the brain and heart following I/R [13–15]. One of our recent studies shows that the activation of TRPV4 promotes intracellular Ca^2+^ entry, increases ROS production, and contributes to myocardial I/R injury [16]. Treatment with the selective TRPV4 antagonist HC-067047 [17] within 12 h after reperfusion has a cardioprotective effect against I/R, as evidenced by a reduced infarct size, decreased troponin T levels, and improved heart function in the *in vivo* model [15]. In addition, Jones et al.'s data confirm that HC-067047 also has a cardioprotective effect in the *ex vivo* Langendorff-perfused heart model [18]. However, further research is required to specify the molecular targets for HC-067047 treatment against myocardial I/R injury.

In this study, we examined the effect of the selective TRPV4 antagonist HC-067047 and TRPV4 siRNA on ROS production, the activity of antioxidative enzymes (SOD, CAT, and GSH-Px), and cellular injury in H9C2 cells subjected to hypoxia/reoxygenation (H/R). To determine whether Akt/Nrf2/ARE signaling is involved in the effects of HC-067047 and TRPV4 siRNA, we pretreated H9C2 cells with the Akt inhibitor LY294002 or used H9C2 cells transfected with Nrf2 siRNA. *Ex vivo* experiments were performed using rat hearts to provide further evidence of the importance of the TRPV4 blockade-induced Akt/Nrf2/ARE pathway in increasing the activity of antioxidative enzymes against oxidative injury following I/R. In addition, the antioxidative effect of HC-067047 was also confirmed in H_2_O_2_-induced myocardial injury. Part of this work has been presented in an abstract form [19].

## 2. Materials and Methods

### 2.1. Cell Isolation and Culture

Rat heart tissue-derived H9C2 cells from ATCC were cultured in Dulbecco's modified Eagle's medium (DMEM, Gibco, USA, #12800), supplemented with 15% fetal bovine serum (FBS, Gibco), 100 U/ml penicillin, and 100 *μ*g/ml streptomycin. The cells were grown in a humidified atmosphere of 95% air and 5% CO_2_ at 37°C. Upon reaching 90% confluency, the cells were passaged with 0.25 mM trypsin/0.03% EDTA. H9C2 cells with >50% subconfluency were transfected with TRPV4-siRNA (5-GGAGCTGAACAAGAACTCA-3, RIBOBIO, Guangzhou, China) or Nrf2-siRNA (Santa Cruz Biotechnology, Dallas, TX, USA, #sc-156128) using Lipofectamine 3000 (Invitrogen). A scrambled nonsilencing siRNA (5-TTCTCCGAACGTGTCACGTd TdT-3, RIBOBIO) was used as a negative control.

To induce H/R injury, cells cultured in a 6-well plate filled with DMEM deficient in glucose and FBS were placed in a controlled hypoxic plastic chamber (HiTech Photoelectricity Biotechnology Co. Ltd., Guangzhou, China) containing 95% N_2_ and 5% CO_2_ for 6 h. Then, the hypoxic medium was replaced by normal DMEM, and cells were cultured in an incubator under a normoxic condition (room air with 5% CO_2_) for 6 h. TRPV4 antagonist HC-067047 (1 *μ*M) and LY294002 (10 *μ*M) were applied at the onset of reoxygenation and hypoxia, respectively. In some experiments, cells were cultured in a Ca^2+^-free medium with 1 mM EGTA.

### 2.2. Animals

Male Sprague-Dawley rats (250-300 g) were individually caged in a constant temperature-controlled (24°C) room, exposed to a 12 h light/dark cycles, and received food and water provided ad libitum. All procedures concerning animal use were performed in adherence to the National Institutes of Health Guide for the Care and Use of Laboratory Animals (eighth edition, 2011) and approved by Tongji Medical College Committee on Animal Care.

Rats were anesthetized by intraperitoneal injection of sodium pentobarbital (40 mg/kg) and heparinized with sodium heparin (125 IU). After thoracotomy, isolated hearts were quickly excised and mounted for retrograde perfusion using a Krebs-Henseleit buffer. The buffer (in mM: NaCl 117, KCl 3.3, KH_2_PO_4_ 1.2, MgSO_4_ 1.2, NaHCO_3_ 24, CaCl_2_ 1.25, D-glucose 11, pH = 7.4) was gassed with 95% O_2_ and 5% CO_2_ at 37°C with a constant flow rate of 10-12 ml/min. A polyethylene water-filled balloon fixed to a pressure transducer was inserted through the mitral valve into the left ventricle to constantly monitor the heart rate (HR), left ventricular end diastolic pressure (LVEDP), left ventricular systolic pressure (LVSP), left ventricle developed pressure (LVDP = LVSP − LVEDP), as well as the maximum increase/decrease rate of left ventricular pressure (±dP/dt max). LVEDP was adjusted to approximately 5 mmHg before the start of the experiment by adjusting the intraventricular balloon volume. The heart rate was set to 420 beats/min by right atrial pacing. Hearts with LVDP < 60 mmHg or >130 mmHg after the stabilization period were discarded. Techman software (Chengdu, China) was used for data acquisition. Hearts were subjected to 30 min of global ischemia followed by 60 min of reperfusion. At the end of the experiment, the hearts were immediately frozen in liquid nitrogen and stored at -80°C until further analysis.

### 2.3. Oxidative Stress, Antioxidant, and Antioxidant Enzyme Activity Determination

The cellular ROS level was evaluated using a DCFH-DA kit (Beyotime Institute of Biotechnology, China, # S0033) as described in the previous study [16]. The fluorescence was detected by a fluorescent microscope or an Enspire Multimode Plate Reader (488 nm excitation and 525 nm emission).

The level of methane dicarboxylic aldehyde (MDA) and the activity of CAT, SOD, and GSH-Px in the heart or H9C2 cell homogenates were determined using an ELISA according to the manufacturer's instructions (Shanghai Enzyme-Linked Biotechnology Co. Ltd., Shanghai, China).

### 2.4. Measurement of Cellular Injury

Cell viability was measured using the Cell Counting Kit-8 (CCK-8, Dojindo Molecular Technologies, Kyushu, Japan) [16]. The optical density at the 450 nm wavelength was measured using a microplate reader (DG5033A, Nanjing, China).

Lactate dehydrogenase (LDH) activity was assessed by an assay kit (Nanjing Jiancheng Biochemistry Co., Nanjing, China) according to the manufacturer's instructions. Coronary effluent samples were collected at the end of reperfusion. Heart sections were stained using triphenyl tetrazolium chloride (TTC) to calculate the percentage of the infarcted area. Infarct size measurement was performed according to previous procedures [15, 16]. The Image-Pro Plus v 6.0 (Media Cybernetics, MD, USA) was used to measure the infarcted area.

### 2.5. Real-Time Quantitative PCR

Total RNA extraction and real-time quantitative PCR were performed according to methods described in previous publications [20]. The primer sequences are listed in Table 1. Data were normalized relative to actin and expressed as a relative ratio. The result for each gene was obtained from at least 6 independent experiments.

### 2.6. Western Blots

Cell lysate preparation and western blot analysis were performed in adherence to established procedures [15, 20]. Whole-cell lysates were obtained using a kit (Servicebio Co. Ltd., Wuhan, China). Nuclear and cytoplasmic fractions were obtained using the Nuclear and Cytoplasmic Protein Extraction Kit (KeyGEN, Nanjing, China). The following primary antibodies were used: Akt (#4691, Cell Signaling), P-Akt (#4060, Cell Signaling), Nrf2 (ab89443, Abcam), TBP (ab51841, Abcam), SOD1 (10269-1-AP, Proteintech), SOD2 (24127-1-AP, Proteintech), CAT (21260-1-AP, Proteintech), GSH-Px1 (#PB0205, BosterBio), and *β*-actin (Servicebio Co. Ltd.). After incubation with horseradish peroxidase-conjugated secondary antibodies, specific bands were visualized by enhanced chemiluminescence using a Bio-Rad ChemiDoc XRS (Bio-Rad, USA) and quantified by Image Lab™ Software.

### 2.7. Immunocytochemistry Assay

Nrf2 expression was detected by immunofluorescence assay. H9C2 cells were cultured in black-walled optically clear-bottomed 96-well plates (Corning Life Sciences, Acton, MA, USA). After the treatment, the cells were incubated with anti-Nrf2 antibodies overnight at 4°C. The antibodies were washed for three 10-minute periods with PBS. The cells were then incubated for 1 h in fluorescein-conjugated secondary antibodies. Subsequently, cell nuclei were stained with DAPI (1 *μ*g/ml for 10 min). The stained cells were washed with PBS and visualized using a fluorescence microscope at 200x magnification.

### 2.8. Statistical Analysis

All values are presented as the mean ± SEM. Data were analyzed by a two-tailed *t-*test or a one-way ANOVA followed by the Bonferroni method analysis. Statistical analysis was performed using SigmaStat3.5 (Systat Software Inc., San Jose, California, USA). Only when *p* < 0.05 was the difference considered statistically significant.

## 3. Results

### 3.1. Effect of TRPV4 Inhibition on ROS and MDA Levels in H9C2 Cells Exposed to H/R

**Figure 1:**
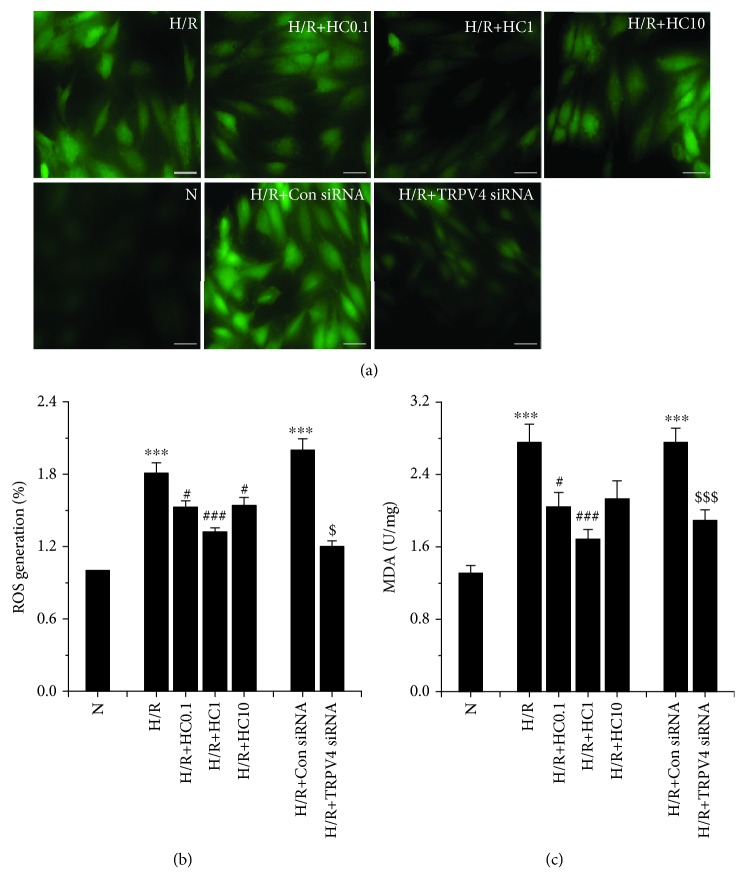
The effect of TRPV4 antagonist HC and TRPV4-siRNA on the levels of ROS and MDA during H/R in H9C2 cells. Images of ROS generation (a) were stained with DCFHDA and generated by fluorescence microscopy. Quantitative analysis of ROS generation (b) was conducted using an Enspire multimode plate reader. MDA levels (c) were measured by the spectrophotometric method. Scale bar: 50 *μ*m. Values are presented as the mean ± SEM; *n* = 6-10/group. We used a one-way ANOVA followed by a Bonferroni test. ^∗∗∗^ *p* < 0.001 vs. N; ^#^ *p* < 0.05 and ^###^ *p* < 0.001 vs. H/R; and ^$^ *p* < 0.05 and ^$$$^ *p* < 0.001 vs. H/R+Con siRNA.

In our previous work, we demonstrated that the activation of TRPV4 induces myocardial injury through increasing oxidative stress during H/R [16]. Thus, we examined whether HC-067047, a TRPV4 specific antagonist, attenuates H/R-induced oxidative stress. The intracellular ROS level was evaluated by measuring DHE staining. Original fluorescent images and the quantitative analysis are shown in Figures 1(a) and 1(b), respectively. The ROS level increase in H/R-induced H9C2 cells compared to cells cultured under a normoxic condition reached a statistical significance (*p* < 0.001). However, HC-067047 (0.1, 1, and 10 *μ*M) treatment significantly inhibited intracellular ROS production. Similarly, HC-067047 reduced MDA levels, and 1 *μ*M HC-067047 had the best protective effect (from 2.76 ± 0.20 U/mg to 1.68 ± 0.11 U/mg, Figure 1(c)). The inhibition of TRPV4 by TRPV4-siRNA transfection also reduces the ROS and MDA levels induced by H/R. These results suggest that the inhibition of TRPV4 reduces the levels of ROS and MDA in H9C2 cells exposed to H/R.

### 3.2. Effect of TRPV4 Inhibition on Antioxidative Enzyme Activity in H9C2 Cells Exposed to H/R

**Figure 2:**
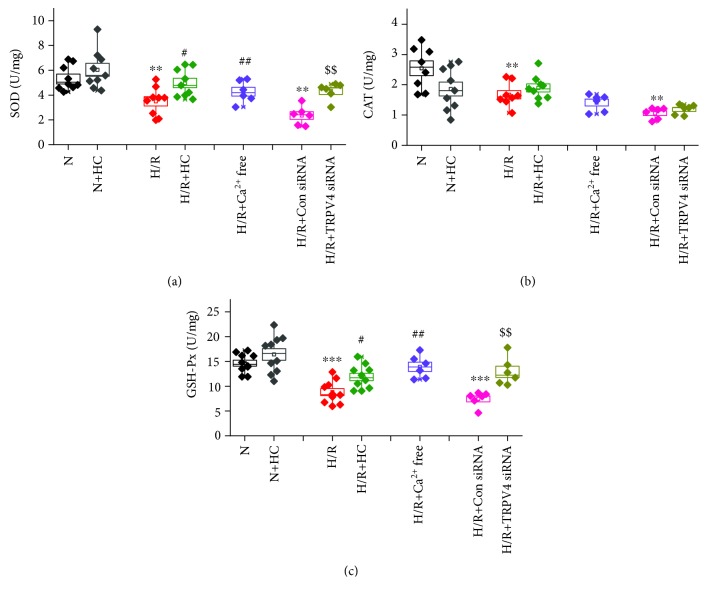
The effect of TRPV4 antagonist HC and TRPV4-siRNA on the activity of SOD (a), GSH-Px (b), and CAT (c) during H/R in H9C2 cells. Values are presented as the mean ± SEM; *n* = 6-10/group. We used a one-way ANOVA followed by a Bonferroni test. ^∗∗^ *p* < 0.01 and ^∗∗∗^ *p* < 0.001 vs. N; ^#^ *p* < 0.05 and ^##^ *p* < 0.01 vs. H/R; and ^$$^ *p* < 0.01 vs. H/R+Con siRNA.

The activity of antioxidant enzyme, including SOD (Figure 2(a)), CAT (Figure 2(b)), and GSH-Px (Figure 2(c)), was significantly reduced in H9C2 cells exposed to H/R. However, the inhibition of TRPV4 by HC-067047 or TRPV4-siRNA greatly increased SOD and GSH-Px activity but had no effect on CAT activity. The activity of SOD and GSH-Px was reversed when the cells were cultured in a Ca^2+^-free medium. However, CAT activity was not affected. This indicates that Ca^2+^ influx via TRPV4 is involved in reducing the activity of SOD and GSH-Px during H/R. In addition, HC-067047 has no effect on the activity of SOD, GSH-Px, and CAT under normoxic conditions. Therefore, the inhibition of TRPV4 enhances the activity of endogenous antioxidant enzymes (particularly SOD and GSH-Px) in H9C2 cells exposed to H/R.

### 3.3. Effect of TRPV4 Inhibition on the Phosphorylation of Akt and the Nuclear Translocation and Levels of Nrf2 in H9C2 Cells Exposed to H/R

**Figure 3:**
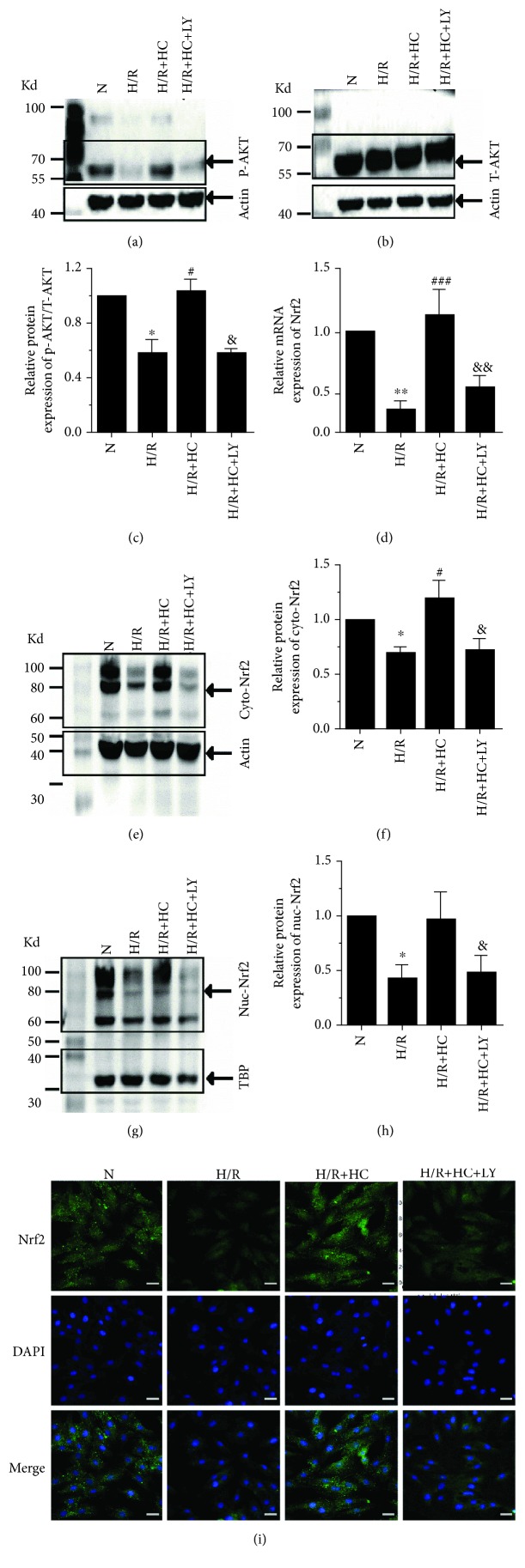
The effect of TRPV4 antagonist HC on Akt/Nrf2 pathway expression in H9C2 cells exposed to H/R. Representative blots of P-Akt (a) and T-Akt (b). A histogram presenting the ratio of P-Akt/T-Akt (c). All of the proteins were normalized to *β*-actin before relative quantitative analysis. The mRNA expression of Nrf2 (d). Representative blots (e) and the histogram (f) of Nrf2 expression in the cytoplasm. Representative blots (g) and the histogram (h) of Nrf2 expression in the nuclei (*n* = 6). The cytoplasmic protein was normalized to *β*-actin, and the nuclei protein was normalized to TBP. Example immunofluorescence images of Nrf2 (i). Values are presented as the mean ± SEM; *n* = 6 for all groups. We used a one-way ANOVA followed by a Bonferroni test. ^∗^ *p* < 0.05 and ^∗∗^ *p* < 0.01 vs. N; ^#^ *p* < 0.05 and ^###^ *p* < 0.001 vs. H/R; and ^&^ *p* < 0.05 and ^&&^ *p* < 0.01 vs. H/R+HC.

To investigate the molecular mechanism underlying the upregulation of antioxidative enzyme activity caused by TRPV4 inhibition, we first assessed the expression of P-Akt and T-Akt (Figures 3(a)–3(c)). We found that H/R could significantly reduce the expression of P-Akt but had no effect on T-Akt. However, the inhibition of TRPV4 by HC-067047 could reverse the reduction of P-Akt induced by H/R. Previous studies show that the phosphorylation of Akt induces Nrf2 transfer to the nucleus and subsequently stimulates the synthesis of several genes encoding antioxidant enzymes [21]. Therefore, we examined the effect of TRPV4 inhibition on Nrf2 expression in the cytoplasm and nucleus using real-time PCR, western blot, and immunofluorescence staining (Figures 3(d)–3(i)). The result was that Nrf2 expression in both the cytoplasm and the nucleus was reduced after H/R. However, inhibiting TRVP4 could prevent this effect. Interestingly, after pretreatment with the Akt inhibitor LY294002, the upregulation of Nrf2 expression in both the cytoplasm and the nucleus in H9C2 cells treated with HC-067047 was significantly attenuated. In addition, the protein level of Keap1, an endogenous Nrf2 inhibitor, markedly decreased in H9C2 cells exposed to H/R, but did not further change after treatment with HC-067047 (Figure S1). These results indicate that the effect of HC-067047 on increasing Nrf2 in H9C2 cells exposed to H/R is related to the activation of Akt, rather than the modulation of Keap1.

### 3.4. Effect of TRPV4 Inhibition on Antioxidant Gene Expression in H9C2 Cells Exposed to H/R

**Figure 4:**
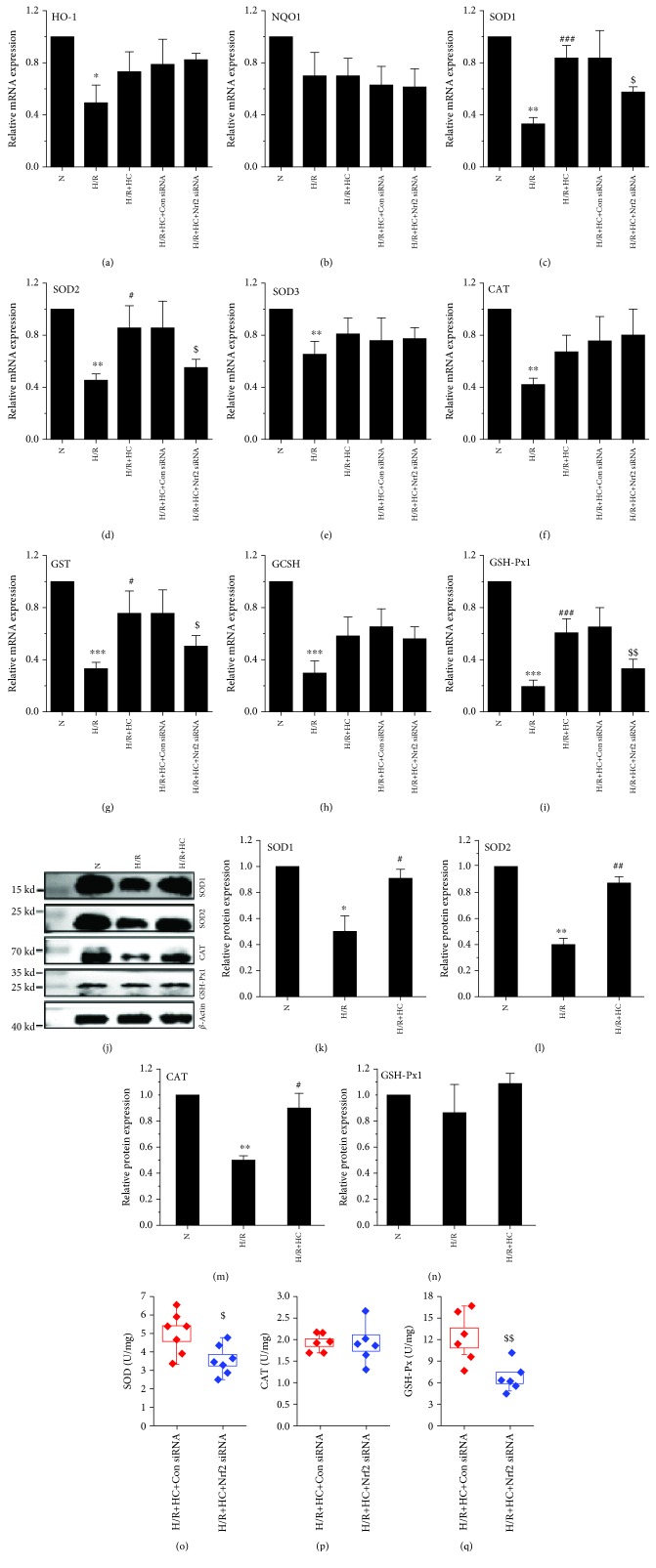
The effect of TRPV4 antagonist HC and TRPV4-siRNA on the expression or activity of antioxidant enzymes in H9C2 cells exposed to H/R. The mRNA expression levels of HO-1, NQO1, SOD1, SOD2, SOD3, CAT, GST, GCSH, and GSH-Px1 were detected by real-time PCR (a–i). The protein levels of SOD1, SOD2, CAT, and GSH-Px1 were examined by western blot (j–n). *β*-Actin was used as an internal control. The activity of SOD (o), GSH-Px (p), and CAT (q) were determined using an ELISA. Values are presented as the mean ± SEM; *n* = 3-6/group. We used a one-way ANOVA followed by a Bonferroni test. ^∗^ *p* < 0.05, ^∗∗^ *p* < 0.01, and ^∗∗∗^ *p* < 0.001 vs. N; ^#^ *p* < 0.05 and ^###^ *p* < 0.001 vs. H/R; and ^$^ *p* < 0.05 and ^$$^ *p* < 0.01 vs. H/R+Con siRNA.

We further measured the mRNA levels of HO-1 (Figure 4(a)), NQO1 (Figure 4(b)), SOD1 (Figure 4(c)), SOD2 (Figure 4(d)), SOD3 (Figure 4(e)), CAT (Figure 4(f)), GST (Figure 4(g)), GCSH (Figure 4(h)), and GSH-Px1 (Figure 4(i)), which are well-known downstream antioxidant molecules of Nrf2 [7]. The expression of HO1, SOD1, SOD2, SOD3, CAT, GST, GCSH, and GSH-Px1 was significantly reduced in H9C2 cells exposed to H/R. However, treatment with HC-067047 increased their expressions. The difference in SOD1, SOD2, GST, and GSH-Px1 was statistically significant while the increased level of HO-1, SOD3, CAT, and GCSH did not reach significance. Protein levels for SOD1 (Figure 4(k)), SOD2 (Figure 4(l)), and CAT (Figure 4(m)) were reduced in H9C2 cells exposed to H/R, and this could be reversed by the pretreatment of H9C2 cells with HC-067047. However, GSH-Px1 protein level was not affected (Figure 4(n)).

To further investigate whether the induction of antioxidant gene expression is directly regulated by Nrf2, we used the siRNA knockdown approach. Nrf2 level was modestly reduced by siRNA in H9C2 cells (data not shown). Remarkably, the depletion of Nrf2 abolished the effect of HC-067047 on increasing mRNA levels of SOD1, SOD2, GST, and GSH-Px1 when compared with the negative control (Figures 4(c), 4(d), 4(g), and 4(i)), further suggesting that the induction of SOD1, SOD2, GST, and GSH-Px1 expression is mainly regulated by the transcriptional activator Nrf2. In addition, the enhancement of endogenous antioxidant enzyme activity was correspondingly reversed (Figures 4(o)–4(q)).

### 3.5. Blocking the PI3K/Akt Pathway or Nrf2 Silencing Reverses the Protective Effect of HC-067047 against H/R-Induced ROS and Injury in H9C2 Cells

**Figure 5:**
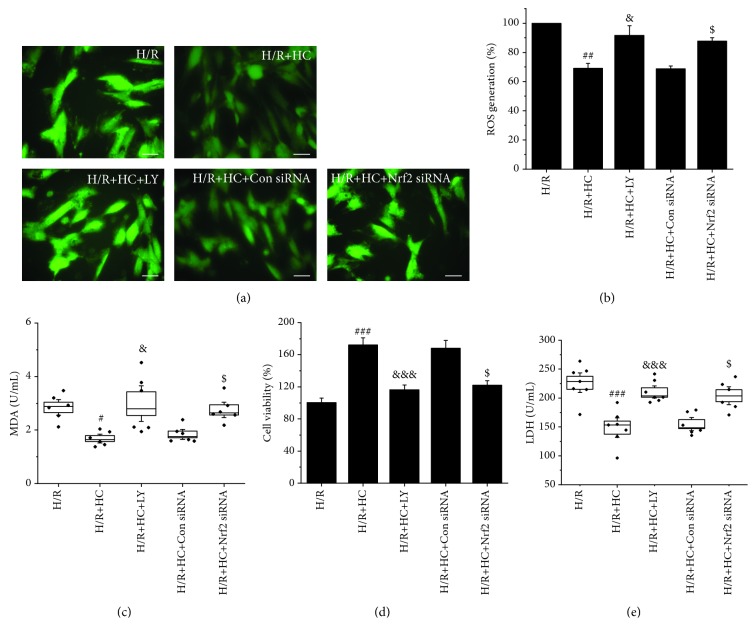
The Akt inhibitor LY294002 and Nrf2 siRNA attenuate the antioxidative and cell protective effects of TRPV4 antagonist HC in H9C2 cells exposed to H/R. Images of ROS generation (a) were stained with DCFHDA and generated by fluorescence microscopy. Quantitative analysis of ROS generation (b) was conducted using an Enspire multimode plate reader. MDA levels were measured by the spectrophotometric method (c). Cell viability was detected by a CCK-8 reduction assay (d). LDH release was measured by LDH assay kits (e). Scale bar: 50 *μ*m. Values are presented as the mean ± SEM; *n* = 6-10/group. We used a one-way ANOVA followed by a Bonferroni test. ^#^ *p* < 0.05, ^##^ *p* < 0.01, and ^###^ *p* < 0.001 vs. H/R; ^&^ *p* < 0.05 and ^&&&^ *p* < 0.001 vs. H/R+HC; and ^$^ *p* < 0.05 and ^$$$^ *p* < 0.001 vs. H/R+Con siRNA.

Subsequently, to determine whether the Akt/Nrf2 pathway is involved in the cardioprotective effect of HC-067047, H9C2 cells were treated with scramble siRNA, Nrf2 siRNA, or LY294002 before HC-067047 and H/R treatment. Later, the levels of oxidative stress (Figures 5(a)–5(c)) and cellular injury (Figures 5(d) and 5(e)) were evaluated. The results demonstrated that pretreatment with Nrf2 siRNA or LY294002 significantly reversed the HC-067047-induced downregulation of ROS, MDA, and LDH levels and the upregulation of cellular viability in H9C2 cells exposed to H/R. However, treatment with scramble siRNA had no effect on ROS, MDA, and LDH levels or cellular viability compared with the HR+HC group. These results suggest that the inhibition of the TRPV4-mediated cardioprotective effect is Akt/Nrf2-dependent.

### 3.6. TRPV4 Antagonists Attenuate Myocardial I/R Injury in Isolated Rat Hearts

**Figure 6:**
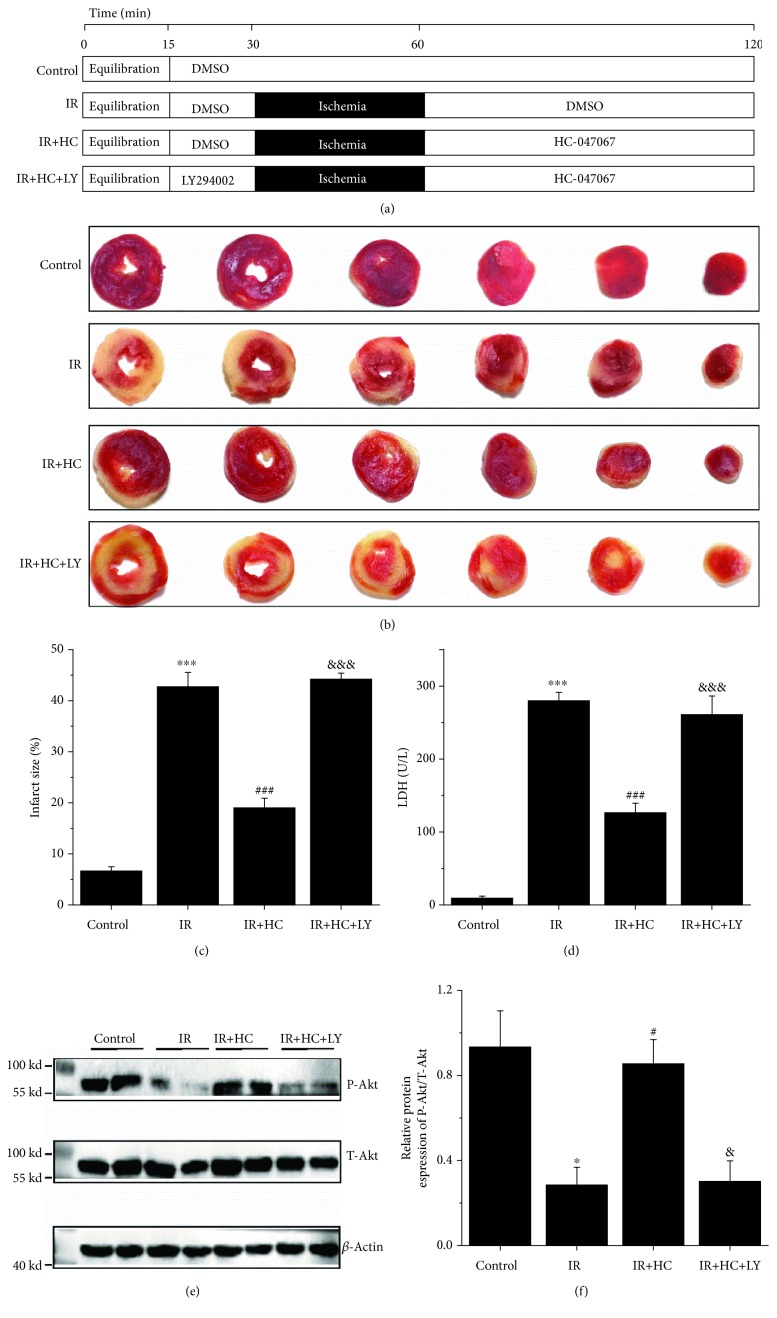
Effects of TRPV4 antagonist HC on myocardial I/R injury in isolated rat hearts. (a) Experimental groups and protocol. (b) Representative images of TTC staining. Red-stained areas represent normal tissue, and unstained pale areas indicate infarcted tissue. (c) Infarct size as a percentage of total volume. (d) The amount of LDH release in coronary effluent at the end of reperfusion. The expression of P-Akt and T-Akt were examined by western blot assay (e) and quantified by densitometric analysis (f). *β*-Actin was used as an internal control. Values are presented as the mean ± SEM; *n* = 6-11 per group, a one-way ANOVA followed by a Bonferroni test. ^∗^ *p* < 0.05 and ^∗∗∗^ *p* < 0.001 vs. control; ^#^ *p* < 0.05 and ^###^ *p* < 0.001 vs. I/R; and ^&^ *p* < 0.05 and ^&&&^ *p* < 0.001 vs. I/R+HC.

The cardioprotective activity of HC-067047 was further tested in isolated rat hearts after I/R injury. Experimental groups and protocol are illustrated in Figure 6(a). As shown in Figures 6(b) and 6(c), TTC staining revealed a marked increase in the infarct size in the I/R group hearts subjected to 30 min of global myocardial ischemia followed by 60 min of reperfusion (from 6.6 ± 0.6% to 43.7 ± 2.1%, *p* < 0.001). However, this effect was attenuated when hearts were treated with HC-067047 (18.9 ± 2.1%, *p* < 0.001 vs. I/R, *p* < 0.001). Furthermore, the analysis of LDH activities in the perfusate (Figure 6(d)) revealed similar trends, and the I/R effects were ameliorated by HC-067047 treatment. We also evaluated cardiac function by continuously monitoring ventricular pressure changes (Table 2). There were no significant differences among the groups during the 15 min of equilibration or before global ischemia (at 30 min). I/R resulted in decreased LVDP and ±dP/dt max at the end of reperfusion (at 120 min) in comparison with the control group. However, HC-067047 treatment significantly attenuated I/R-induced myocardial dysfunction in the reperfusion period. Again, pretreatment with the Akt inhibitor LY294002 could abolish the cardioprotective effect of HC-067047 in isolated rat hearts. Simultaneously, a western blot assay was employed to evaluate the levels of P-Akt/Akt (Figures 6(e) and 6(f)). The results showed that HC-067047 treatment markedly reversed I/R-induced downregulation of Akt phosphorylation. The data suggest that TRPV4 inhibition could indeed reduce myocardial I/R injury mainly through the activation of Akt.

### 3.7. TRPV4 Antagonists Reduce I/R-Induced Oxidative Stress in Isolated Rat Hearts

**Figure 7:**
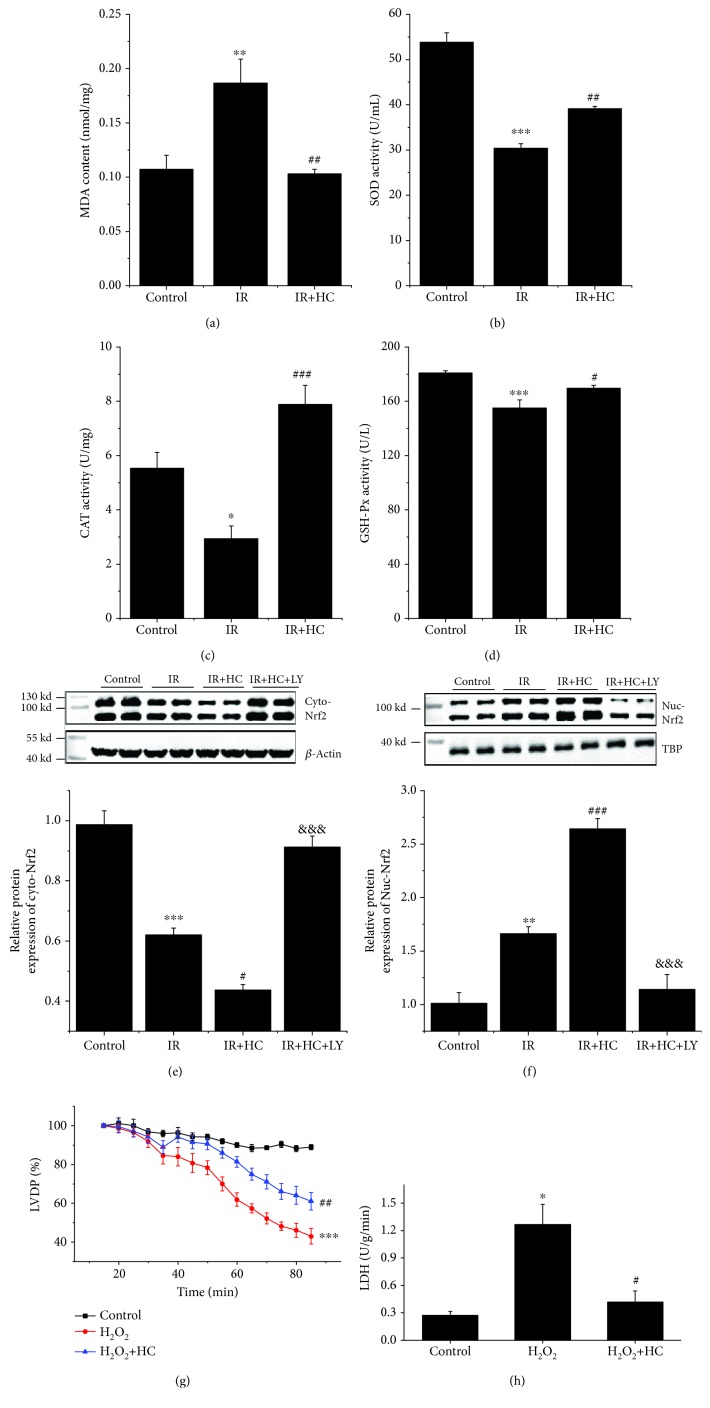
Effects of TRPV4 antagonist HC on attenuating I/R-Induced oxidative stress in isolated rat hearts. (a) Production of MDA in heart tissue. The activity of SOD (b), GSH-Px (c), and CAT (d) in heart tissue. (d) Representative blots and the histogram of Nrf2 expression in the cytoplasm (e) and nuclei (f). The cytoplasmic protein was normalized to *β*-actin, and the nuclei protein was normalized to TBP. (g) LVDP during perfusion with H_2_O_2_ (100 *μ*M) displayed as the percentage of respective stabilization values. (h) The amount of LDH release in coronary effluent at the end of experiment. Values are presented as the mean ± SD; *n* = 6 per group, a one-way ANOVA followed by a Bonferroni test; ^∗^ *p* < 0.05, ^∗∗^ *p* < 0.01, and ^∗∗∗^ *p* < 0.001 vs. control; ^#^ *p* < 0.05, ^##^ *p* < 0.01, and ^###^ *p* < 0.001 vs. I/R or H_2_O_2_; and ^&&&^ *p* < 0.001 vs. I/R+HC.

Later, the antioxidant capacity of HC-067047 was examined in isolated rat hearts. As shown in Figure 7(a), the MDA levels in the I/R group were significantly higher than those in the control group. In contrast, SOD, CAT, and, GSH-Px activities in the I/R group were markedly lower than those in the control group (Figures 7(b)–7(d)). Interestingly, HC-067047 treatment could significantly diminish the MDA levels as well as increase SOD, CAT, and GSH-Px activities. Consistent with previous research, I/R remarkably reduced cytosol Nrf2 expression but significantly increased the nuclear level of Nrf2 [22]. Also, HC-067047 could further reduce cytosol Nrf2 and increase nuclear Nrf2, suggesting that HC-067047 could activate Nrf2 to translocate to the nuclei. However, pretreatment with LY294002 significantly reversed the HC-067047-induced Nrf2 nuclear translocation effect (Figures 7(e) and 7(f)). We also investigated the effect of HC-067047 on H_2_O_2_-induced myocardial injury. After 15 min of equilibration, hearts were retroperfused with H_2_O_2_ (100 *μ*M) for 70 min. Compared with the control group, H_2_O_2_ induced a gradual but significant decrease in LVDP (Figure 7(g)). HC-067047 treatment significantly inhibited the effect of H_2_O_2_ on LVDP (Figure 7(g)). Similarly, LDH levels were markedly reduced by HC-067047 treatment (Figure 7(h)). We performed the western blot assay to evaluate the expression of P-Akt, T-Akt, Keap1, Nrf2, and SOD (Figure S2). H_2_O_2_ induced significant decreases in the P-Akt/T-Akt, Nrf2, and SOD1 expressions and marked increases in Keap1 expressions compared to the data in control. Treatment with HC could reverse these H_2_O_2_-induced alterations. Our findings strongly indicate that the inhibition of TRPV4 has a cardioprotective effect against oxidative stress *ex vivo*, which is closely related to the activation of the Akt/Nrf2/ARE pathway.

## 4. Discussion

The present study examined the antioxidative effect of TRPV4 inhibition against myocardial I/R injury and assessed the underlying mechanisms. The results of the current study demonstrate that treatment with the selective TRPV4 antagonist HC-067047 or TRPV4-siRNA transfection attenuates the intracellular ROS and MDA levels, improves the activity and expressions of intracellular antioxidant enzymes, and ultimately reverses H/R-induced cytotoxicity in H9C2 cells. Moreover, HC-067047 and TRPV4-siRNA both increase the phosphorylation of Akt and the activation of Nrf2/ARE signaling. Blocking the Akt pathway or Nrf2 silencing reverses the antioxidative action of HC-067047 *in vitro*. Furthermore, we used isolated rat hearts to confirm that treatment with HC-067047 can improve cardiac function and reduce LDH release during I/R or H_2_O_2_. In addition, the blockade of TRPV4 can effectively reduce oxidative stress levels, increase the activity of intracellular antioxidant enzymes, and activate the Akt/Nrf2/ARE pathway *ex vivo*.

Oxidative stress plays a central role in the pathogenesis of myocardial I/R-induced injury [23]. Antioxidant enzymes, including SOD, GSH-Px, and CAT, protect cells from oxidative damage [8]. As an efficient enzyme, SOD catalyzes the conversion of superoxide into O_2_ and H_2_O_2_. Then, CAT and GSH-Px further decompose H_2_O_2_ into H_2_O and O_2_. Consistent with the previous studies, H/R enhances ROS production and apoptosis of H9C2 cells and reduces the activity and expression of SOD, GSH-Px, and CAT [24–27]. However, treatment with HC-067047 or TRPV4-siRNA transfection reverses H/R-induced reduction of SOD and GSH-Px activity. Moreover, similar with TRPV4 inhibition, the removal of extracellular Ca^2+^ also abolishes H/R-induced SOD and GSH-Px activity reduction in H9C2 cells, indicating that Ca^2+^ influx via TRPV4 channels is involved in the regulation of SOD and GSH-Px activity. Although H/R-induced reduction of CAT activity was not affected by treatment with HC-067047, the mRNA and protein levels of this enzyme were reversed. In fact, the activation of TRPV4 has been shown to downregulate the activity of antioxidant enzymes in the hippocampus [28]. In addition, treatment with HC-067047 could upregulate SOD, GSH-Px, and CAT activity and downregulate the MDA level in isolated hearts exposed to I/R. These findings suggest that the inhibition of TRPV4 could mitigate oxidative stress by enhancing the antioxidant activity and expressions in the myocardium after I/R.

The induction of SOD, GSH-Px, and CAT gene transcription is highly dependent on the conserved oligonucleotide sequence ARE. Although several transcription factors bind to ARE, Nrf2 is the key regulator of the antioxidant defense mechanism against myocardial I/R injury [8]. Increasing evidence indicates that numerous cardioprotective drugs can alleviate I/R-induced oxidative stress by upregulating Nrf2/ARE activation [29–31]. In our research, we found that treatment with HC-047067 or TRPV4-siRNA promotes the nuclear translocation of Nrf2 and increases Nrf2 expression after myocardial I/R injury. In addition, antioxidant enzyme genes (SOD1, SOD2, GST, and GSH-Px) are upregulated in H9C2 cells treated with HC-047067 or TRPV4-siRNA. These effects of TPPV4 inhibition are abrogated by Nrf2 silencing. These findings clearly indicate that TRPV4 inhibition-induced Nrf2 activation plays an important role in alleviating oxidative stress in H9C2 cells exposed to H/R. However, future research is required to determine whether TRPV4 inhibition induces transcription factors other than Nrf2, which regulates antioxidant enzyme gene transcription [32].

Akt has been found to be an important regulator in myocardial I/R injury [33, 34]. Consistent with our previous *in vivo* study [15], the inhibition of TRPV4 by HC-047067 or TRPV4-siRNA promotes the phosphorylation of Akt and alleviates myocardial injury both *in vitro* and *ex vivo*. Simultaneously, we observed the upregulation of the mRNA and protein levels of SOD. In cardiomyocytes, the upregulation of SOD stimulates mitochondrial H_2_O_2_ production, which in turn activates Akt [35]. Therefore, we assumed that Akt activation by the inhibition of TRPV4 might have occurred through the SOD/H_2_O_2_ pathway. However, the underlying mechanisms require further research. Nrf2 has been shown to be a downstream target of Akt [36]. The activation of Akt promotes Nrf2 phosphorylation and thereby activates Nrf2 nuclear translocation, which subsequently upregulates the expression of antioxidant genes by binding to ARE [36]. The increase in Nrf2 nuclear translocation and Nrf2 expression, as well as the upregulation of the expression of antioxidant enzyme genes (SOD1, SOD2, GST, and GSH-Px) by TRPV4 inhibition, is apparently reversed by the Akt inhibitor LY294002. The results demonstrate that the activation of Akt is associated with Nrf2 activation in H9C2 cells treated with HC-067047 or TRPV4-siRNA. The activation of the Akt/Nrf2/ARE signaling pathway attenuates the generation of ROS and the apoptosis of cardiomyocytes, showing a promising cardioprotective effect against myocardial I/R injury [37]. We showed that the Akt inhibitor LY294002 or Nrf2 siRNA nearly canceled the cardioprotective effects of TRPV4 inhibition, providing potent evidence that Akt/Nrf2/ARE acts as a cytoprotective signaling mechanism for TRPV4 inhibition.

## 5. Conclusions

**Figure 8:**
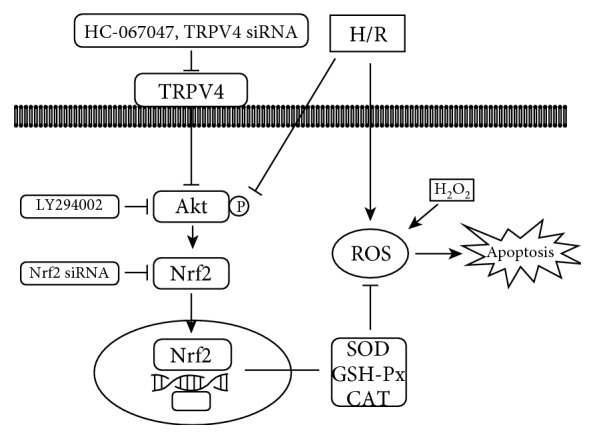
This scheme shows the potential mechanisms of TRPV4 inhibition against myocardial I/R injury by suppression the oxidative stress via the Akt/Nrf2/ARE pathway.

This study confirms our previous finding that TRPV4 inhibition effectively protects against myocardial I/R injury and that this effect is likely mediated by the upregulation of the Akt/Nrf2/ARE-dependent antioxidant defense system (Figure 8). This is a potential mechanism for the antioxidative action of TRPV4 inhibition.

## Figures and Tables

**Table 1:** Primers used for real-time PCR.

Gene	Primer sequences
HO-1	Forward: AGGTGCACATCCGTGCAGAG
	Reverse: CTTCCAGGGCCGTATAGATATGGTA
NQO1	Forward: TGGAAGCTGCAGACCTGGTG
	Reverse: CCCTTGTCATACATGGTGGCATAC
SOD1	Forward: AACCAGTTGTGGTGTCAGGA
	Reverse: CTCCTGAGAGTGAGATCACA
SOD2	Forward: TTCTGGACAAACCTGAGCCCTAA
	Reverse: GAACCTTGGACTCCCACAGACAC
SOD3	Forward: AGGCTCTTTCTCAGGCCTC
	Reverse: AGATCTCCAGGTCTTTGGAG
CAT	Forward: GCGAATGGAGAGGCAGTGTAC
	Reverse: GAGTGAGTTGTCTTCATTAGCACTG
GST	Forward: TTCGTGCAGACATTGTGGAGA
	Reverse: CTTGCCCAGGAACTCAGAGTAGA
GCSH	Forward: CACCGGATCTGCTTTGCTCTC
	Reverse: TTGCTGATTCCCACTGTTCCAATA
GSH-Px1	Forward: AGGAGAATGGCAAGAATGAAGAGA
	Reverse: GGAAGGTAAAGAGCGGGTGAG
*β*-Actin	Forward: CGTTGACATCCGTAAAGACC
	Reverse: TAGAGCCACCAATCCACACA

**Table 2:** Hemodynamic parameters of isolated rat hearts subjected to global ischemia/reperfusion.

	LVDP (mmHg)	+dP/dt max (mmHg/s)	-dP/dt max (mmHg/s)	LVDP (% preischemia)	+dP/dt max (% preischemia)	-dP/dt max (% preischemia)
C, *n* = 7						
15	75.1 ± 9.2	4523.3 ± 492.0	- 3414.3 ± 224.4			
30	71.6 ± 6.6	4799.3 ± 382.8	- 3356.8 ± 181.6			
120	67.1 ± 7.7	4473.4 ± 536.5	- 3145.6 ± 198.3	93.8 ± 6.1	93.1 ± 7.5	93.8 ± 5.5
I/R, *n* = 11						
15	69.1 ± 7.6	4339.4 ± 464.1	- 3197.1 ± 271.2			
30	67.3 ± 10.4	4449.8 ± 635.6	- 3454.1 ± 358.1			
120	12.2 ± 2.8^∗∗∗^	2206.8 ± 223.3^∗∗∗^	- 2287.1 ± 260.1^∗∗∗^	18.3 ± 3.7^∗∗∗^	50.3 ± 7.3^∗∗∗^	66.7 ± 4.9^∗∗∗^
I/R+HC, *n* = 6						
15	70.7 ± 5.6	4070.8 ± 684.4	- 3322.8 ± 281.5			
30	65.3 ± 9.4	3945.2 ± 784.8	- 3110.5 ± 146.5			
120	40.7 ± 7.5^###^	3017.8 ± 273.2^###^	- 2616.1 ± 169.8^#^	62.9 ± 12.3^###^	78.2 ± 12.4^###^	84.1 ± 4.0^###^
IR+HC+LY, *n* = 7						
15	69.1 ± 7.6	4770.2 ± 519.9	- 3408.7 ± 250.0			
30	71.3 ± 13.8	4582.1 ± 646.6	- 3315.0 ± 285.1			
120	13.4 ± 5.9^&&&^	2376.2 ± 148.9^&&^	- 2308.3 ± 184.9	18.3 ± 6.5^&&&^	52.7 ± 7.7^&&&^	70.0 ± 7.3^&&&^

Parameters measured after stabilization period (15 min), prior to ischemia (30 min), and at the end of reperfusion (120 min). C: control group; I/R: ischemia/reperfusion; HC: HC-067047; LY: LY294002; LVDP: left ventricular developed pressure; ±dP/dt max: the maximum increase/decrease rate of left ventricular pressure. Values are presented as the mean ± SD; *n* = 6-11/group. We used a one-way ANOVA followed by a Bonferroni test. ^∗^ *p* < 0.05, ^∗∗^ *p* < 0.01, and ^∗∗∗^ *p* < 0.001 vs. C; ^#^ *p* < 0.05, ^##^ *p* < 0.01, and ^###^ *p* < 0.001 vs. I/R; and ^&^ *p* < 0.05, ^&&^ *p* < 0.01, and ^&&&^ *p* < 0.001 vs. I/R.

## Data Availability

The data used to support the findings of this study are available from the corresponding authors upon request.

## Supplementary Materials

Supplementary Materials Figure S1: the effect of TRPV4 antagonist HC on Keap1 expression in H9C2 cells exposed to H/R. The expression of Keap1 was examined by western blot assay (A) quantified by densitometric analysis (B). *β*-Actin was used as an internal control. Values are presented as the mean ± SEM; *n* = 3 for all groups. We used a one-way ANOVA followed by a Bonferroni test. ^∗∗^ *p* < 0.01 and ^∗∗∗^ *p* < 0.001 vs. N. Figure S2: the effect of TRPV4 antagonist HC on Akt, Keap1, Nrf2, SOD1, and SOD2 expression in isolated hearts exposed to H_2_O_2_. Representative blots (A) and the histogram of the expression of P-Akt/T-Akt (B), Keap1 (C), Nrf2 (D), SOD1(E), and SOD2 (F). *β*-Actin was used as an internal control. Values are presented as the mean ± SEM; *n* = 6 for all groups. We used a one-way ANOVA followed by a Bonferroni test. ^∗∗∗^ *p* < 0.001 vs. control; ^###^ *p* < 0.001 vs. H_2_O_2_.
