# Supplementary material for: Blockade of Transient Receptor Potential Vanilloid 4 Enhances Antioxidation after Myocardial Ischemia/Reperfusion
Source: Oxid Med Cell Longev. 2019 Jun 16;2019:7283683. doi: 10.1155/2019/7283683 (PMC6604422; doi:10.1155/2019/7283683)
Supplement: Supplementary Materials — Figure S1: the effect of TRPV4 antagonist HC on Keap1 expression in H9C2 cells exposed to H/R. The expression of Keap1 was examined by western blot assay (A) quantified by densitometric analysis (B). β-Actin was used as an internal control. Values are presented as the mean ± SEM; n = 3 for all groups. We used a one-way ANOVA followed by a Bonferroni test. ∗∗ p < 0.01 and ∗∗∗ p < 0.001 vs. N. Figure S2: the effect of TRPV4 antagonist HC on Akt, Keap1, Nrf2, SOD1, and SOD2 expression in isolated hearts exposed to H2O2. Representative blots (A) and the histogram of the expression of P-Akt/T-Akt (B), Keap1 (C), Nrf2 (D), SOD1(E), and SOD2 (F). β-Actin was used as an internal control. Values are presented as the mean ± SEM; n = 6 for all groups. We used a one-way ANOVA followed by a Bonferroni test. ∗∗∗ p < 0.001 vs. control; ### p < 0.001 vs. H2O2. [file 7283683.f1.pdf]

Supplementary Data

Figure S1

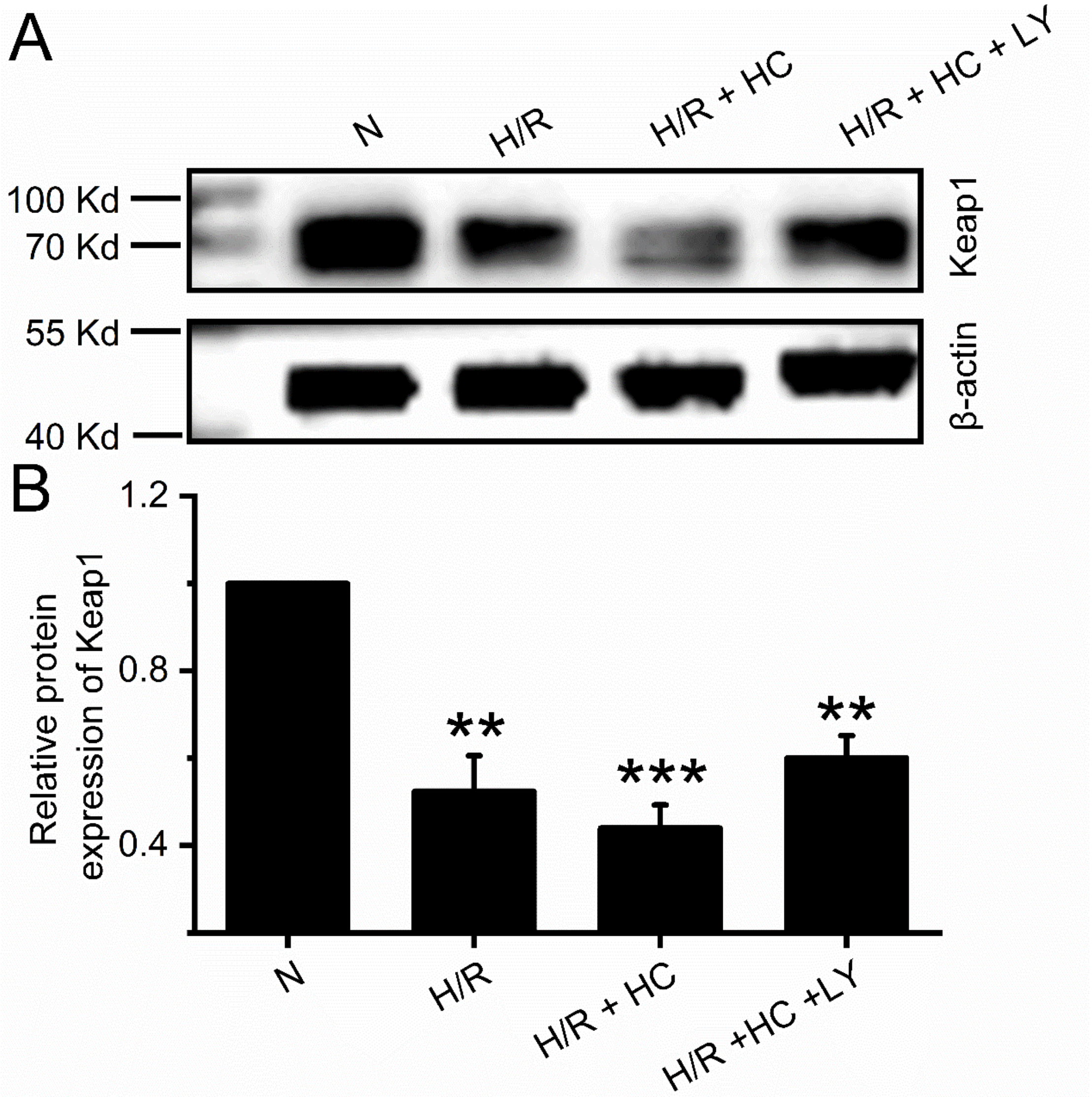

Figure S1. The effect of TRPV4 antagonist HC on Keap1 expression in H9C2 cells exposed to H/R. The expression of Keap1 was examined by Western blot assay (A) quantified by densitometric analysis (B). β-actin was used as an internal control. Anti-Keap1 antibody was purchased from Proteintech (10503-2-AP). Values are presented as mean ± SEM, n = 3 for all groups. We used a one-way ANOVA followed by a Bonferroni test. ** $p < 0.01$, *** $p < 0.001$ vs. N.

Figure S2

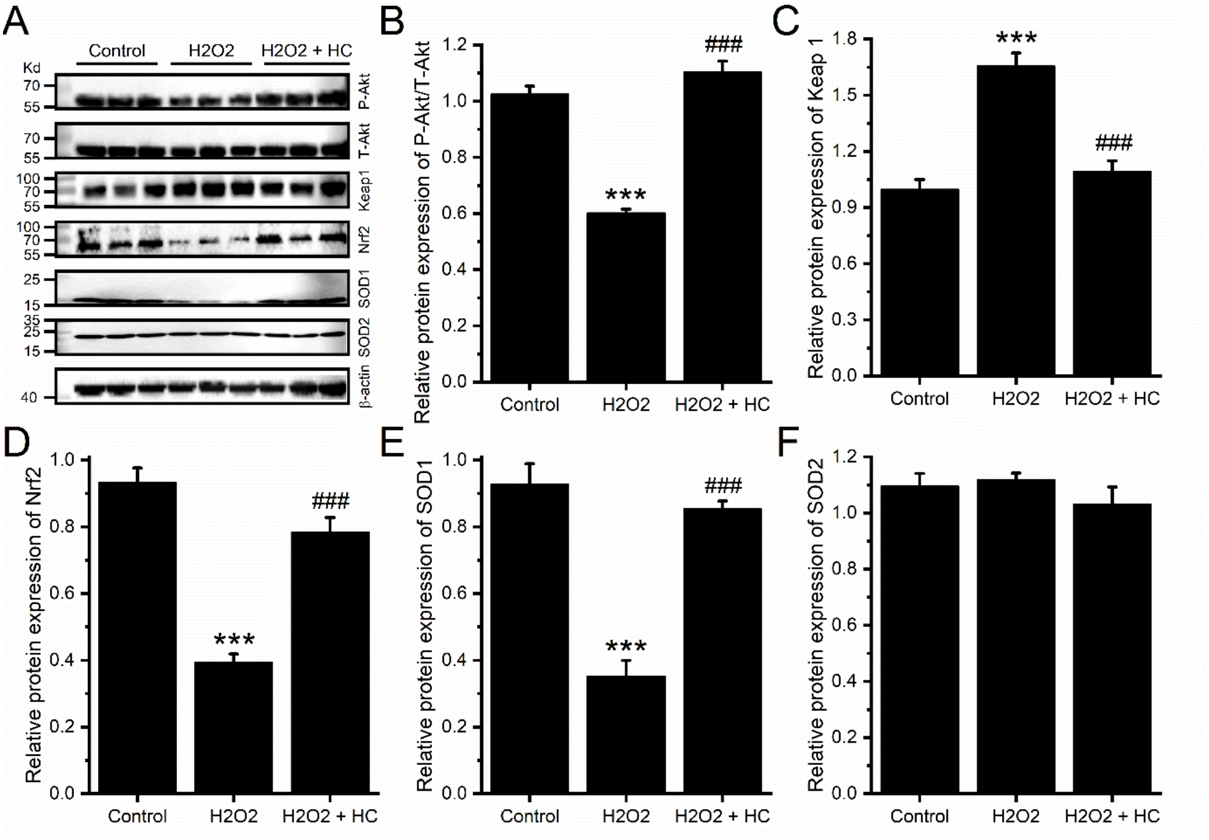

Figure S2. The effect of TRPV4 antagonist HC on Akt, Keap1, Nrf2, SOD1, and SOD2 expression in isolated hearts exposed to H_2O_2. Representative blots (A) and the histogram of the expression of P-Akt/T-Akt (B), Keap1 (C), Nrf2 (D), SOD1(E), and SOD2 (F). β-actin was used as an internal control. Values are presented as mean ± SEM, n = 6 for all groups. We used a one-way ANOVA followed by a Bonferroni test. *** $p < 0.001$ vs. control; ### $p < 0.001$ vs. H_2O_2.
